# Development of a quantitative segmentation model to assess the effect of comorbidity on patients with COVID-19

**DOI:** 10.1186/s40001-020-00450-1

**Published:** 2020-10-12

**Authors:** Cui Zhang, Guangzhao Yang, Chunxian Cai, Zhihua Xu, Hai Wu, Youmin Guo, Zongyu Xie, Hengfeng Shi, Guohua Cheng, Jian Wang

**Affiliations:** 1 Department of Radiology, TongDe Hospital of ZheJiang Province, No.234, Gucui Road, Hangzhou, 310012 Zhejiang China; 2 Department of Radiology Center, The Second People’s Hospital of Neijiang, Number 470, Road Xinjing, Neijiang, Sichuan China; 3 Department of Radiology, Wenzhou People’s Hospital, Number 57, Road Canghou, Wenzhou, Zhejiang China; 4 Department of Radiology, The First Affiliated Hospital of Xi’an Jiaotong University, Xi’an, Shanxi China; 5 Department of Radiology, The First Affiliated Hospital of Bengbu Medical College, Number 287, Road Changhuai, Bengbu, Anhui China; 6 Department of Radiology, Anqing Municipal Hospital, Number 352, Road Renmin, Anqing, Anhui China; 7 Jianpei Technology, Hangzhou, Zhejiang China

**Keywords:** Comorbidity, COVID-19, Deep learning, X-ray computed tomography

## Abstract

**Background:**

The coronavirus disease 2019 (COVID-19) has brought a global disaster. Quantitative lesions may provide the radiological evidence of the severity of pneumonia and further to assess the effect of comorbidity on patients with COVID-19.

**Methods:**

294 patients with COVID-19 were enrolled from February, 24, 2020 to June, 1, 2020 from six centers. Multi-task Unet network was used to segment the whole lung and lesions from chest CT images. This deep learning method was pre-trained in 650 CT images (550 in primary dataset and 100 in test dataset) with COVID-19 or community-acquired pneumonia and Dice coefficients in test dataset were calculated. 50 CT scans of 50 patients (15 with comorbidity and 35 without comorbidity) were random selected to mark lesions manually. The results will be compared with the automatic segmentation model. Eight quantitative parameters were calculated based on the segmentation results to evaluate the effect of comorbidity on patients with COVID-19.

**Results:**

Quantitative segmentation model was proved to be effective and accurate with all Dice coefficients more than 0.85 and all accuracies more than 0.95. Of the 294 patients, 52 (17.7%) patients were reported having at least one comorbidity; 14 (4.8%) having more than one comorbidity. Patients with any comorbidity were older (*P* < 0.001), had longer incubation period (*P* < 0.001), were more likely to have abnormal laboratory findings (*P* < 0.05), and be in severity status (*P* < 0.001). More lesions (including larger volume of lesion, consolidation, and ground-glass opacity) were shown in patients with any comorbidity than patients without comorbidity (all *P* < 0.001). More lesions were found on CT images in patients with more comorbidities. The median volumes of lesion, consolidation, and ground-glass opacity in diabetes mellitus group were largest among the groups with single comorbidity that had the incidence rate of top three.

**Conclusions:**

Multi-task Unet network can make quantitative CT analysis of lesions to assess the effect of comorbidity on patients with COVID-19, further to provide the radiological evidence of the severity of pneumonia. More lesions (including GGO and consolidation) were found in CT images of cases with comorbidity. The more comorbidities patients have, the more lesions CT images show.

## Background

The coronavirus disease 2019 (COVID-19) caused by severe acute respiratory syndrome coronavirus 2 (SARS-CoV-2) broke out in Wuhan, China in December 2019. It has widely spread all over the world, which has led to a major concern [1–3]. Up to July 28, 2020, 16,341,920 and 650,805 confirmed and death cases have been reported worldwide. The United States, as the largest epicenter, had the confirmed and death cases of 4,209,509 and 146,331, respectively [4].

A sharp increase in the number of cases due to human-to-human transmission has resulted in high rates of hospitalization and intensive care unit (ICU) admission, which caused an extreme shortage of medical resources [5]. Therefore, it is especially important to use limited medical resources and social support for people with serious condition and prone to serious outcomes.

Previous reports found that comorbidity (including hypertension, diabetes mellitus, respiratory system disease, and cardiovascular) may be a risk factor of COVID-19 progression and also correlated with poorer clinical outcomes [6–10]. The relationship between COVID-19 and comorbidity was mostly explored from a clinical perspective, so far few studies have studied it in radiology.

Chest CT has played a pivotal diagnostic role in the assessment of the disease severity according to the number, extent, density of patchy ground-glass opacities (GGOs), and consolidation [11]. Differential diagnosis, severity rating, and prognosis prediction about COVID-19 have been investigated using a quantitative CT combined with artificial intelligence (AI) technology [12–14]. However, quantitative CT study about the effect of comorbidity on patients with COVID-19 has not been reported, which may provide the radiological evidence of the severity of pneumonia.

In our study, the multi-task Unet, a deep learning method, was used to develop a segmentation model to quantify the pneumonia lesion including the volume of GGOs and consolidation to assess the effect of comorbidity on patients with COVID-19.

## Materials and methods

### Patients

This was a retrospective study that collected data from six centers (TongDe Hospital of ZheJiang Province, The Second People's Hospital of Neijiang, The First Affliated Hospital of Bengbu Medical College, Wenzhou People's Hospital, Anqing Municipal Hospital, and The First Affiliated Hospital of Xi'an Jiaotong University). This study was approved by the Ethics Committee of the above hospitals, and written informed consents were waived, because the anonymized study did not alter any diagnosis and treatment of the patients. 294 patients were enrolled from 24, February, 2020 to 1, June, 2020. We included patients who satisfied the following criteria: (a) positive for next­generation sequencing or real­time RT­PCR of SARS-CoV-2 in throat or nose swabs; (b) complete clinical data; (c) patients underwent CT scans. The exclusion criteria were (a) poor images with heavy breathing artifacts or metal artifacts; (b) patients had history of pulmonary surgery. This research was limited to the confirmed COVID-19, not including suspicious or negative cases. When the patient had series of CT examinations, the most severe sets of images were included in our study.

### Clinical information

The basic data including gender, age, incubation period, symptom, comorbidity (hypertension, diabetes mellitus, cardiovascular disease, cerebrovascular disease, COPD, hepatitis B infection, malignancy, chronic kidney disease, and immunodeficiency), severity status (severe or non-severe), laboratory examinations including C reactive protein (CRP), white blood cell count (WBC), and lymphocyte count were extracted from medical computerized database for all patients. Incubation period was defined as the interval between the potential date of transmission source (suspected or confirmed cases) contacts and the date of symptom onset (i.e., fever, cough, fatigue, dyspnea, and myalgia). For the patient who had a history of travel in epidemic area, incubation period was defined as the interval between the date of entry into or exit from that place and the date of symptom onset. The severity of COVID-19 includes four types: mild, common, severe, and critical according to the guideline of 2019-nCoV (trial version ^7^) issued by the China National Health Commission [15]. In this study, we divided all the patients into severe group (including severe and critical) with 38 cases and non-severe group (mild and common) with 256 cases. Laboratory examinations were collected at the time of admission or within two days.

### CT image acquisition

The non-contrast chest CT scans were performed using three multi-detector CT scanners with 64 or 128 channels (Somatom Definition AS+, Siemens Healthineers, Forchheim, Germany; GE Medical Systems, China Branch, Beijing, China or Philips Ingenuity Core128, Philips Medical Systems, Best, The Netherlands). The scanning range was from apex to the base of lungs. The detailed parameters for CT acquisition were as follows: tube voltage, 120 kVp; tube current, standard (reference mAs, 60–120) to low-dose (reference mAs, 30) with automatic exposure control; slice thickness, 1.0 or 1.25 mm; reconstruction interval, 1.0–3.0 mm; noise index (NI), 25; and matrix 512 × 512. A lung window was with a width of 2000 HU and a level of − 600HU, and a mediastinal window with a width of 350 HU and a level of 40HU. All patients underwent first CT scans within 0–3 days of admission. 231 (78.6%) patients had multiple CT scans.

### CT image segmentation and quantitative analysis

The deep learning method used in our study is Unet neural network [16] which has been reported to have a good performance on the segmentation of the biomedical images. Here, we used a multi-task Unet with a single encoder and two parallel decoders to learn to predict and segment the region of lung and lesions. The decoder containing attention block was used to learn to segment the lesions, while the decoder containing stacked dilated convolutions was used to learn the lung segmentation, which provided a more efficient feature encoding and a regularizing effect. This neural network were implemented in Dr. Pecker cloud platform (https://www.jianpeicn.com/category/yuepianjiqiren) and our segmentation results were acquired from the platform. Our platform is open and free to all public research institutions in the world.

**Figure1:**
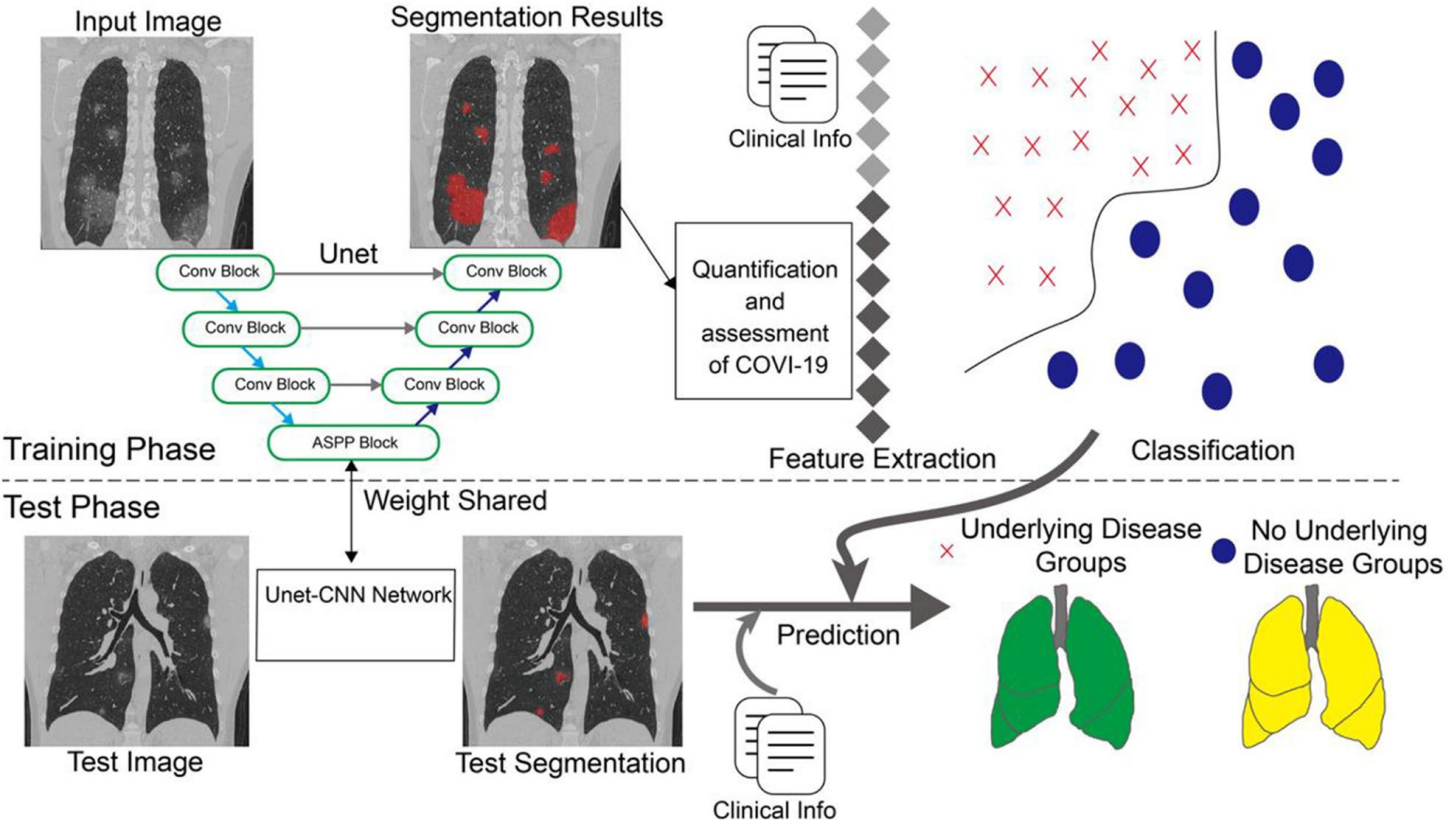
The general flow of Unet neural network to segment lung and lesions. Our neural network model was trained in the training dataset, and tested on test dataset. 550 CT images were split into primary dataset and 100 were primary dataset, respectively. First, CT images were inputted into this neural network to extract image features, segment lung, and lesion, and further classify whether the lesion was consolidation or GGO. The outputted results were the volumes of the lesions in underlying disease group and no underlying disease group

To make the neural network to learn to predict lesion and lung regions, labeled lesion and lung samples were required. The lesion and lung regions were manually segmented using ITK-SNAP software (version 2.2.0; https://www.itksnap.org) in lung window with a width of 2000 HU and a level of − 600 HU. Our segmentation system was pre-trained by 650 annotated CT images (550 in primary dataset and 100 in test dataset) with COVID-19 or community-acquired pneumonia. This neural network extracted CT image features, segmented lung and lesions, and classified whether the lesion was consolidation or GGO. The volumes of the lesions as the results in underlying disease group and non-underlying disease group were outputted finally. The general flow of this study is shown in Fig. 1.

**Figure2:**
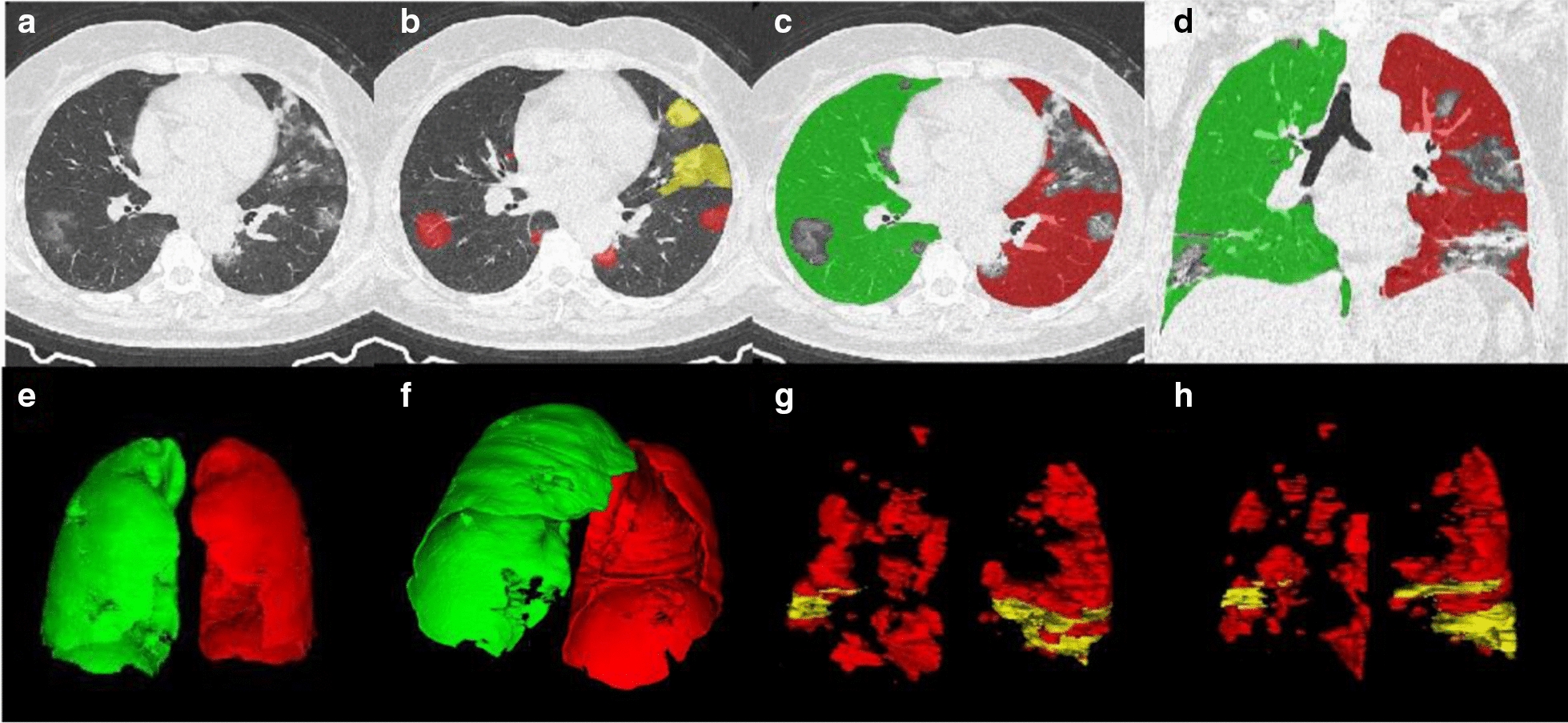
Novel coronavirus pneumonia in a 50-year-old woman in diabetes mellitus group with fever and cough for 8 days before admission. **a** Axial plane of CT scan in the lung window showed multiple irregular pieces of GGO (black arrow) and consolidation (white arrow). **b** GGO (red area) and consolidation (yellow area) were identified and marked according to value of − 450 HU. **c**, **d** Axial and coronal planes of CT image showed the margin of the lesion in each slice was delineated. **e**, **f** A 3D lung erosion diagram exhibited the regions of lesion erosion (white arrows). **g**, **h** A 3D lesion diagram demonstrated the volume of GGO (red area) and consolidation (yellow area)

**Figure3:**
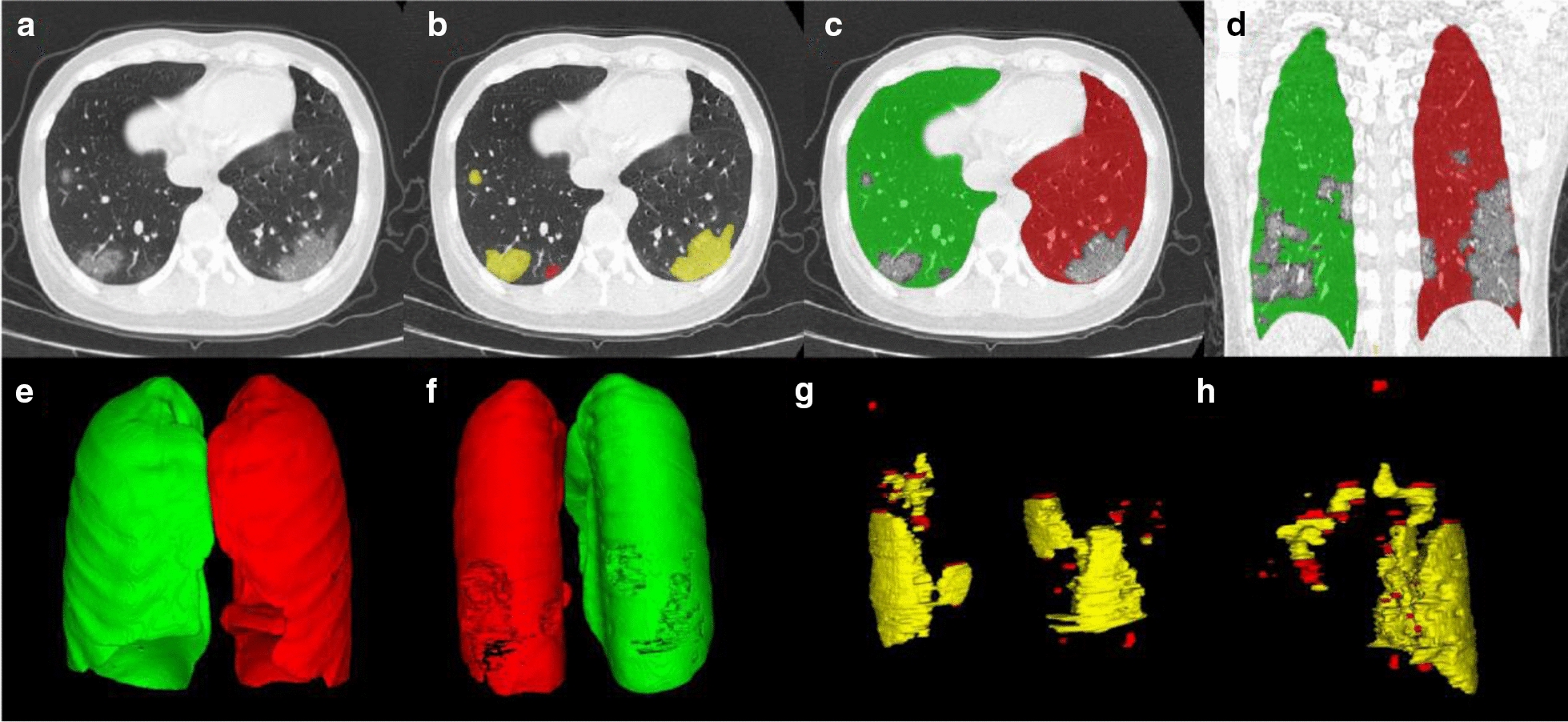
Novel coronavirus pneumonia in a 25-year-old woman without underlying disease. **a**, **b** Axial plane of CT scan in the lung window showed most lesions were GGO (red area) with a little consolidation (yellow area) under the pleura. **c**, **d** The margin of the lesion was delineated showed in axial and coronal planes of CT image. **e**–**h** A 3D lung erosion diagram exhibited the regions of lesion erosion (white arrows). **g**, **h** A 3D region of lesion (white arrows in **e**, **f**) was obtained based on delineated results including lung erosion diagram (**e**, **f**) and lesion diagram (**g**, **h**)

The specific image segmentation steps were as follows. First, a threshold value of − 450 HU was used to distinguish GGO and consolidation (Figs. 2, 3b). The margin of the lesion in each axial slice was delineated (Figs. 2, 3c, d). Then, a 3D region of interest (ROI) was obtained based on delineated results including lung erosion diagram (Figs. 2, 3e, f) and lesion diagram (Figs. 2, 3g, h). The SimpleITK software tool (https://www.simpleitk.org) was used to quantify the mean HU of lung and lesions, volumes, and numbers of lesions automatically.

Dice coefficient (between 0 and 1) in test dataset was used as an index to evaluate the quantitative segmentation effect of this model. Higher Dice coefficient presents better model.

To assess the accuracy of segmentation on our cohort, two cardiothoracic radiologists (C.Z. and J.W. with 5 and 12 years of experience, respectively) manually marked the lesion area of 50 CT scans of 50 patients (15 with comorbidity and 35 without comorbidity) and compared the results with those of segmentation model.

In total, the following eight quantitative parameters were acquired for further analysis: (a) the volume of the whole lung (LUV); (b) the volume of lesion (LEV); (c) the ratio of volume of lesion to whole lung (LEV/LUV); (d) the volume of consolidation (COV); (e) the ratio of volume of consolidation to whole lung (COV/LUV); (f) the volume of GGO (GGOV); (g) the ratio of volume of GGO to whole lung (GGOV/LUV); and (h) the number of lesions (LEN).

## Statistical analysis

Continuous variables were presented as median (IQR) and categorical variables were shown as *n* (%). Mann–Whitney *U* test, Chi-square test, or Fisher’s exact test were used to compare differences between patients with and without comorbidity. Statistical analyses were performed with SPSS (ver. 22.0; SPSS Inc., Chicago, IL, USA). Two-sided* P* < 0.05 was considered statistically significant.

## Results

### Patients with or without comorbidity

Of all the patients, there were 52 patients with comorbidity and the remaining 242 patients without comorbidity. Among these patients with comorbidity, 34 (65.4%) patients were with hypertension, 15 (28.9%) with diabetes mellitus, 8 (15.4%) with hepatitis B infection, 4 (7.7%) with cardiovascular disease, 3 (5.8%) with COPD, and 2 (3.8%) with cerebrovascular disease. Patient with malignancy, chronic kidney disease, and immunodeficiency was 1 (1.9%).

### Segmentation effect of this model

The average Dice coefficient in test dataset was 0.973 for the right lung, 0.985 for left lung, and 0.864 for lesion segments (all > 0.85), suggesting the good performance of our neural network in lung and lesion segmentation task. The accuracy of segmentation was 0.987 for all lesions, 0.968 for consolidation, and 0.953 for GGOs (all > 0.95).

### Characteristics of patients with or without any comorbidity

Of the 294 cases, 52 (17.7%) patients were reported having at least one comorbidity. Patients with any comorbidity were older (median: 55.50 vs. 44.00 years) (*P* < 0.001), had longer incubation period (median: 6.00 vs. 3.00 days) (*P* < 0.001). They were more likely to have abnormal CPR (75.0% vs. 53.7%) (*P* < 0.01), WBC (32.7% vs. 18.6%) (*P* < 0.05), and lower lymphocyte count (0.58 vs. 0.72) (*P* < 0.01), and be in severity status (42.3% vs. 6.6%) (*P* < 0.01). Patients with comorbidities had more possibility to have dyspnea and more than one symptom (both *P* < 0.01) (Table 1). As for the quantitative CT images analysis, larger LEV (median: 235.01 vs. 127.82 cm^3^) (*P* < 0.001), LEV/LUV (median: 0.07 vs. 0.04) (*P* < 0.001), COV (median: 94.58 vs. 40.45 cm^3^) (*P* < 0.001), COV/LUV (median: 0.03 vs. 0.01) (*P* < 0.01), GGOV (median: 150.26 vs. 74.71 cm^3^) (*P* < 0.001), GGOV/LUV (median: 0.04 vs. 0.02) (*P* < 0.001), and LEN (median: 30.00 vs. 19.00) (*P* < 0.01) were shown in patients with at least one comorbidity than patients without comorbidity. The difference in gender distribution was not significant between two groups (Table 1).

**Table 1:** Characteristics of patients with or without any comorbidity

Variables	Total (*n* = 294)	Without comorbidity (*n* = 242)	With any comorbidity (*n* = 52)	*P* value
Gender				0.149
Male	134 (45.6%)	127 (52.5%)	19 (36.5%)	
Female	160 (54.4%)	115 (47.5%)	33 (63.5%)	
Age (years)	46.00 (34.75–54.00)	44.00 (32.00–53.00)	55.50 (47.00–65.25)	*0.000*
Incubation period (days)	5.00 (3.00–7.00)	3.00 (2.00–7.00)	6.00 (4.00–8.00)	
Symptom				
Fever	275 (93.4%)	223 (92.2%)	52 (100%)	0.075
Dyspnea	170 (57.8%)	129 (53.3%)	41 (78.9%)	*0.001*
More than one symptom	225 (76.5%)	178 (73.6%)	47 (90.4%)	*0.009*
CRP abnormal				*0.005*
Yes	169 (57.5%)	130 (53.7%)	39 (75.0%)	
No	125 (42.5%)	112 (46.3%)	13 (25.0%)	
WBC abnormal				*0.024*
Yes	62 (21.1%)	45 (18.6%)	17 (32.7%)	
No	232 (78.9%)	197 (81.4%)	35 (67.3%)	
LY count ( × 10^9^/L)	0.63 (0.45–1.13)	0.72 (0.51–1.12)	0.58 (0.35–0.93)	*0.001*
Yes	135 (45.9%)	101 (41.7%)	34 (65.38%)	
No	159 (54.1%)	141 (58.3%)	18 (34.62%)	
Severity status				*0.000*
Yes	38 (12.9%)	16 (6.6%)	22 (42.3%)	
No	256 (87.1%)	226 (93.4%)	30 (57.7%)	
Quantitative CT analysis				
LUV (cm^3^)	3728.28 (2944.22–4642.55)	3709.28 (2939.74–4538.04)	4180.53 (3027.12–4868.62)	0.218
LEV (cm^3^)	139.25 (58.28–297.34)	127.82 (50.50–265.91)	235.01 (89.65–658.18)	*0.000*
LEV/LUV	0.04 (0.02–0.09)	0.04 (0.01–0.08)	0.07 (0.02–0.16)	*0.000*
COV (cm^3^)	45.00 (12.97–119.82)	40.45 (11.82–96.27)	94.58 (23.93–208.55)	*0.000*
COV/LUV	0.01 (0.00–0.04)	0.01 (0.00–0.03)	0.03 (0.02–0.06)	*0.001*
GGOV (cm^3^)	80.02 (36.95–169.48)	74.71 (31.08–150.72)	150.26 (50.36–305.02)	*0.000*
GGOV/LUV	0.02 (0.00–0.05)	0.02 (0.01–0.04)	0.04 (0.02–0.09)	*0.000*
LEN	22.00 (11.00–39.00)	19.00 (10.00–37.25)	30.00 (21.25–44.75)	*0.001*

CRP: C reactive protein; WBC: white blood cell count; LY: lymphocyte; LUV: the volume of the whole lung; LEV: the volume of lesion; COV: the volume of consolidation; GGOV: the volume of GGO; LEN: the number of lesions

*P* values written in italic indicate a significant difference

### Characteristics of patients with 1 comorbidity or ≥ 2 comorbidities

Of the 52 patients with any comorbidity, 38 (73.1%) patients have one comorbidity and the remaining 14 (26.9%) had more than one comorbidity. Older age (median: 53.00 vs. 44.00 and 60.00 vs. 44.00 years), longer incubation period (median: 6.00 vs. 3.00 and 8.00 vs. 3.00 days), more likely to have lower lymphocyte count (0.64 vs. 0.72 and 0.45 vs. 0.72), and more likely in severe state (36.8% vs. 6.6% and 57.1vs. 6.6%) were exhibited both in cases with 1 comorbidity or ≥ 2 comorbidities compared to cases without any comorbidity. In terms of quantitative analysis of CT imaging, both patients with 1 comorbidity and more than one comorbidity were detected more lesions (LEV: 235.87 vs. 127.82 cm^3^ and 237.17 vs. 127.82 cm^3^; LEV/LUV: 0.07 vs. 0.04 and 0.09 vs. 0.04; COV: 97.86 vs. 40.45 cm^3^ and 94.89 vs. 40.45 cm^3^; COV/LUV: 0.03 vs. 0.01 and 0.04 vs. 0.01; GGOV: 135.80 vs. 74.71 cm^3^ and 197.58 vs. 74.71 cm^3^; GGOV/LUV: 0.03 vs. 0.02 and 0.05 vs. 0.02). More tendencies to have abnormal CPR and more numbers of lesions were found in patients with one comorbidity, whereas there was no difference between patients with ≥ 2 comorbidities and without comorbidity. Neither patients with one comorbidity nor with ≥ 2 comorbidities have significant difference in gender distribution and abnormal WBC count (Table 2).

**Table 2:** Characteristics of patients with 1 comorbidity or ≥ 2 comorbidities

Variables	Without comorbidity (*n* = 242)	1 comorbidity (*n* = 38)	≥ 2 comorbidities (*n* = 14)	*P*1 value	*P*2 value
Gender				0.126	0.734
Male	127 (52.5%)	13 (34.2%)	6 (42.9%)		
Female	115 (47.5%)	25 (65.8%)	8 (57.1%)		
Age (years)	44.00 (32.00–53.00)	53.00 (47.00–61.00)	60.00 (52.00–67.00)	*0.000*	*0.000*
Incubation period (days)	3.00 (2.00–7.00)	6.00 (4.00–7.00)	8.00 (3.00–13.00)	*0.029*	*0.041*
Symptoms					
Fever	223 (92.2%)	38 (100%)	14 (100%)	0.149	0.572
Dyspnea	129 (53.3%)	29 (76.3%)	12 (85.7%)	*0.008*	0.065
More than one symptom	178 (73.6%)	35 (92.1%)	12 (85.7%)	0.479	0.486
CRP abnormal				*0.021*	0.072
Yes	130 (53.7%)	28 (73.7%)	11 (78.6%)		
No	112 (46.3%)	10 (26.3%)	3 (21.4%)		
WBC abnormal				0.138	0.064
Yes	45 (18.6%)	11 (28.9%)	6 (42.9%)		
No	197 (81.4%)	27 (71.1%)	8 (57.1%)		
LY count (× 10^9^/L)	0.72 (0.51–1.12)	0.64 (0.39–0.94)	0.45 (0.27–0.89)	*0.043*	*0.036*
Severity status				*0.000*	*0.000*
Yes	16 (6.6%)	14 (36.8%)	8 (57.1%)		
No	226 (93.4%)	22 (42.3%)	6 (42.9%)		
Quantitative CT analysis					
LUV (cm^3^)	3709.28 (2939.74–4538.04)	3885.28 (2846.22–4916.78)	4170.63 (3129.45–4697.75)	0.606	0.252
LEV (cm^3^)	127.82 (50.50–265.91)	235.87 (85.14–629.24)	237.17 (161.81–837.53)	*0.001*	*0.009*
LEV/LUV	0.04 (0.01–0.08)	0.07 (0.02–0.15)	0.09 (0.04–0.27)	*0.001*	*0.017*
COV (cm^3^)	40.45 (11.82–96.27)	97.89 (23.65–200.86)	94.86 (24.80–289.90)	*0.001*	*0.022*
COV/LUV	0.01 (0.00–0.03)	0.03 (0.01–0.06)	0.04 (0.01–0.11)	*0.001*	*0.046*
GGOV (cm^3^)	74.71 (31.08–150.72)	135.80 (49.43–281.20)	197.58 (66.95–377.93)	*0.003*	*0.006*
GGOV /LUV	0.02 (0.01–0.04)	0.03 (0.02–0.10)	0.05 (0.02–0.15)	*0.003*	*0.013*
LEN	19.00 (10.00–37.25)	31.00 (20.00–46.00)	30.00 (23.00–32.00)	*0.001*	0.202

CRP: C reactive protein; WBC: white blood cell count; LY: lymphocyte; LUV: the volume of the whole lung; LEV: the volume of lesion; COV: the volume of consolidation; GGOV: the volume of GGO; LEN: the number of lesions

*P*1 value: 1 comorbidity vs. without comorbidity; *P*2 value: ≥ 2 comorbidities vs. without comorbidity; *P* values written in italics indicate a significant difference

### Characteristics of patients with different comorbidities

To assess the impact of a certain underlying disease with a higher incidence on COVID-19, we separately compared the clinical and imaging indicators of these patients. Of the 52 patients with comorbidities, 22 (16.9%), 7 (3.7%), and 8 (1.9%) patients were reported only having hypertension, diabetes, and hepatitis B infections, that had the incidence rate of top three. These three series all have more possibility in severity status compared with series without comorbidity (all *P* < 0.001). Patients in hypertension group had more possibility to have dyspnea and more than one symptom (both *P* < 0.05), and they were more likely to have lower lymphocyte count (*P* < 0.05). It was worth noting that almost all quantitative indicators except GGOV and LEN indicated that patients with hypertension and diabetes mellitus had more lesions than patients without any comorbidity. However, there were no differences between patients with hepatitis B infections and without comorbidity in almost all clinical and quantitative CT characteristics except lower lymphocyte count (Table 3).

**Table 3:** Characteristics of patients with different comorbidities

Variables	Without comorbidity (*n* = 242)	Hypertension (*n* = 22)	Diabetes mellitus (*n* = 7)	Hepatitis B infection (*n* = 8)	*P*1 value	*P*2 value	*P*3 value
Gender					0.552	0.544	0.111
Male	127 (52.5%)	13 (59.1%)	5 (71.4%)	7 (87.5%)			
Female	115 (47.5%)	9 (40.9%)	2 (28.6%)	1 (12.5%)			
Age (years)	44.00 (32.00–53.00)	56.00 (48.50–62.25)	51.00 (43.00–69.00)	46.00 (43.00–52.00)	*0.000*	0.055	0.315
Incubation period (days)	3.00 (2.00–7.00)	6.50 (4.00–7.25)	6.00 (5.00–7.00)	4.00 (2.25–6.50)	0.076	0.208	0.835
Symptoms							
Fever	223 (92.2%)	22 (100%)	7 (100%)	8 (100%)	0.351	1.000	1.000
Dyspnea	129 (53.3%)	19 (86.4%)	6 (85.7%)	4 (50.0%)	*0.003*	0.190	1.000
More than one symptom	178 (73.6%)	21 (95.5%)	6 (85.7%)	6 (75.0%)	*0.022*	0.775	1.000
CRP abnormal					*0.003*	1.000	0.586
Yes	130 (53.7%)	19 (86.4%)	4 (57.1%)	3 (37.5%)			
No	112 (46.3%)	3 (13.6%)	3 (42.9%)	5 (62.5%)			
WBC abnormal					0.773	*0.041*	0.379
Yes	45 (18.6%)	3 (13.6%)	4 (57.1%)	3 (37.5%)			
No	197 (81.4%)	19 (86.4%)	3 (42.9%)	5 (62.5%)			
LY count (× 10^9^/L)	0.72 (0.51–1.12)	0.60 (0.31–0.89)	0.69 (0.56–1.10)	0.56 (0.35–0.94)	*0.044*	0.977	*0.039*
Severity status					*0.000*	*0.000*	*0.010*
Yes	16 (6.6%)	7 (31.8%)	3 (42.9%)	3 (37.5%)			
No	226 (93.4%)	15 (68.2%)	4 (57.1%)	5 (62.5%)			
Pleural effusion					1.000	0.859	0.935
Yes	13 (5.4%)	1 (4.5%)	1 (14.3%)	1 (12.5%)			
No	229 (94.6%)	21 (95.5%)	6 (85.7%)	7 (87.5%)			
Quantitative CT analysis							
LUV (cm^3^)	3709.28 (2939.74–4538.04)	3279.78 (2647.81–4775.08)	4289.78 (3043.78–5644.50)	4457.41 (2895.61–5104.92)	0.545	0.400	0.393
LEV (cm^3^)	127.82 (50.50–265.91)	192.55 (68.09–581.92)	570.00 (206.43–1365.34)	187.85 (76.33–361.09)	*0.036*	*0.001*	0.315
LEV/LUV	0.04 (0.01–0.08)	0.05 (0.02–0.15)	0.17 (0.05–0.26)	0.04 (0.02–0.09)	*0.033*	*0.001*	0.429
COV (cm^3^)	40.45 (11.82–96.27)	74.24 (21.58–212.75)	178.87 (119.98–605.27)	67.43 (21.00–168.81)	*0.039*	*0.001*	0.345
COV/LUV	0.01 (0.00–0.03)	0.02 (0.01–0.06)	0.04 (0.03–0.11)	0.02 (0.00–0.05)	*0.045*	*0.001*	0.390
GGOV (cm^3^)	74.71 (31.08–150.72)	104.19 (46.51–212.08)	369.13 (66.95–765.53)	82.82 (48.20–169.62)	0.075	*0.004*	0.471
GGOV/LUV	0.02 (0.01–0.04)	0.03 (0.02–0.08)	0.10 (0.02–0.15)	0.02 (0.01–0.04)	*0.047*	*0.003*	0.590
LEN	19.00 (10.00–37.25)	37.00 (20.00–46.25)	37.00 (22.00–46.00)	24.50 (16.75–45.25)	*0.004*	0.068	0.190

CRP: C reactive protein; WBC: white blood cell count; LY: lymphocyte; LUV: the volume of the whole lung; LEV: the volume of lesion; COV: the volume of consolidation; GGOV: the volume of GGO; LEN: the number of lesions

*P*1 value: hypertension vs. without comorbidity; *P*2 value: diabetes mellitus vs. without comorbidity; *P*3 value: hepatitis B infection vs. without comorbidity; *P* values written in italics indicate a significant difference

## Discussion

In this study, we tried a multi-task Unet network, a segmentation tool to build a quantitative segmentation model to assess the impact of comorbidity on patients with COVID-19. Comorbidity may be a risk factor for severe status and pneumonia progression. Furthermore, they are also associated to poor clinical outcomes which consists of admission to ICU, or invasive ventilation, or death [6–10]. However, most previous studies were focused on the relationship between COVID-19 and comorbidity from a clinical perspective. As a radiologist with a very important position during the outbreak, we first assessed the impact of comorbidity with intuitive and quantitative data. It is of great necessity to provide the radiological evidence of the severity of pneumonia before treatment, which may greatly determine the clinical management and prognosis.

Our neural network model was trained in primary dataset, and tested on test dataset to confirm the robustness of it. The average Dice coefficients in test dataset were all more than 0.85 for lung and lesion segments, which suggested good performance in lung and lesion segmentation task. The lesion regions of all 294 cases were segmented by our multi-task Unet network first, and then, a part of segmentation results was checked manually. More than 95% lesion regions were segmented accurately, which indicated stability and accuracy of our Unet model segmentation.

From the quantitative CT images analysis, larger lesion volumes (including consolidation and GGOs) were found in patients with any comorbidity than without comorbidity. It is worth taking note of that the more comorbidity patients have, the more lesions CT images show. The more the lung parenchymal is involved, the more severe condition it would be. The appearance of GGO indicates that alveolar cavity is partially filled by fluid and cells, while the appearance of consolidation demonstrates that further accumulation of exudates in alveolar cavity and aggravation of interstitial edema, which always means disease progresses [17]. The increasing numbers of GGOs and densities of consolidation always indicate disease deterioration [18].

In our study, patients with underling diseases were older, more likely to have abnormal CPR, WBC, and lower lymphocyte count. They tended to have worse condition, dyspnea, and more than one symptom, simultaneously. Age, comorbidities, and lymphopenia had been shown to be associated with progression, poor prognosis, and increased mortalities [19–21]. More WBC count, more CRP level, and lower lymphocyte count were found in patients in severe status and in the ICU [22, 23]. Reports above indicated that abnormal laboratory finding, severe condition, and comorbidity may predict poor outcomes.

The pathogenesis of COVID-19 may be associated with underlying diseases due to their susceptibility conditions. Researchers reported that similar results were found in MERS [24]. Comorbidities are characterized by proinflammatory state and the attenuation of the immune response [19, 25]. For instance, a possible cause of diabetes mellitus is the accumulation of activated innate immune cells in metabolic tissues leading to the release of inflammatory mediators, thereby promoting insulin resistance and β-cell damage [26]. Furthermore, metabolic disorders may lead to decreased immune function because of impaired macrophage and lymphocyte function [27]. Impaired immune function eventually may make patients more susceptible to other diseases [24]. Furthermore, High expression of angiotensin converting enzyme 2 (ACE2) in diabetes and hypertension may potentially facilitate viral uptake [28, 29]. Our study found that patients with diabetes mellitus has the worst CT findings; however, the specific mechanism still remains in-depth study. In addition, patients with more than one comorbidity have greater possibility to have impaired immune function, which may be linked to more severe lung CT images.

Unexpectedly, we found that neither consolidation nor GGO volumes were not significantly different when comparing patients with hepatitis B infections and without comorbidity, although they were more likely in severity status. This may suggest that the severity of COVID-19 is not completely consistent with the CT findings. Meta-analyses from Wang et al. [9] and Lippi et al. [30] did not provide sufficient evidence that liver disease was relevant to COVID-19 progress. However, the credibility of our results may be limited by the small number of cases with liver disease.

To our surprise, the results revealed that incubation period of individuals with comorbidity was longer than that of patients without comorbidity, which may have relationship with the insensitivity reaction to COVID-19 in comorbidity groups. The rapid progresses of pneumonia and more lesions showed on CT may be partly on account of delayed timely treatment.

This study had several limitations. First of all, small sample sizes of patients with a certain underlying disease alone may affect the credibility of some results and conclusions. Second, the most severe CT images included in our study cannot show the change of the COVID-19 in patients with or without comorbidities, which can prompt us to investigate and study in the next-step. Third, the novelty of the algorism of the quantitative assessment model may not be very outstanding compared to previously reported ones.

## Conclusions

In conclusion, multi-task Unet network can make quantitative CT analysis of lesions to assess the effect of comorbidity on patients with COVID-19. More lesions (including GGO and consolidation) were found in CT images of cases with comorbidity. The more comorbidities patients have, the more lesions CT images manifest.

## Data Availability

All data generated or analyzed during this study are included in this published article.
